# Spermatic Cord Liposarcoma: A Case Report and Review of the Literature on the Role of Radiotherapy and Chemotherapy in Preventing Locoregional Recurrence

**DOI:** 10.7759/cureus.19567

**Published:** 2021-11-14

**Authors:** Olisaemeka D Ogbue, Abdo Haddad, Hamed Daw

**Affiliations:** 1 Internal Medicine, Cleveland Clinic Fairview Hospital, Cleveland, USA; 2 Hematology and Oncology, Cleveland Clinic Fairview Hospital, Cleveland, USA

**Keywords:** wide resection, spermatic cord tumors, chemotherapy, radiotherapy (rt), well-differentiated liposarcoma

## Abstract

Spermatic cord cancer is a rare entity. Among malignant tumors of the spermatic cord, liposarcomas are the most common type, often presenting as painless slow-growing masses usually in the fifth and sixth decades of life; they can be misdiagnosed as inguinal hernia or hydrocele. Radical orchiectomy with wide local soft tissue resection is an accepted standard of care for spermatic cord liposarcoma and has been curative in some cases. There is no definitive role for other treatment modalities such as chemotherapy, retroperitoneal lymph node dissection (RPLND), and radiotherapy. We present a case of liposarcoma of the spermatic cord managed with radical orchiectomy, wide local excision, and was followed up without disease recurrence. We also engage in a review of the literature on the role of systemic chemotherapy and radiotherapy in preventing locoregional recurrence after primary surgery.

A combination of surgery and postoperative radiotherapy is effective in preventing locoregional spread. Data from case reports support this strategy in certain histologic subtypes or when margins are positive after primary surgery. A follow-up period of up to a decade after surgery is recommended.

## Introduction

Spermatic cord cancer is a rare entity. Primary paratesticular tumors account for 7-10% of all intrascrotal tumors [1]. These tumors are categorized based on their site of origin within the scrotum such as testicular tunica, epididymis, or spermatic cord. The spermatic cord is the most common site of occurrence in adults, accounting for 75% of primary paratesticular tumors [1]. Among malignant tumors of the spermatic cord, liposarcomas are the most common type. They often present as painless slow-growing masses usually in the fifth and sixth decades of life and can be misdiagnosed as inguinal hernia or hydrocele.

Radical orchiectomy with wide local soft tissue resection is an accepted standard of care for spermatic cord liposarcoma and has been curative in some cases [2]. The probability of local recurrence after excision is reportedly high due to the high-grade nature of liposarcoma of the spermatic cord. There is no definitive role for other treatment modalities such as chemotherapy, retroperitoneal lymph node dissection (RPLND), and radiotherapy. Despite being radiosensitive, the role of radiotherapy in its management remains controversial. While it is believed to help in reducing local recurrence, the overall treatment outcome remains unclear. Clear management guidelines for pre or postoperative radiotherapy are lacking due to the paucity of data.

We present a case of liposarcoma of the spermatic cord managed with radical orchiectomy, wide local excision, and was followed up without disease recurrence. We also review the literature on the role of systemic chemotherapy and radiotherapy in preventing the locoregional recurrence of this rare entity.

## Case presentation

**Figure 1:**
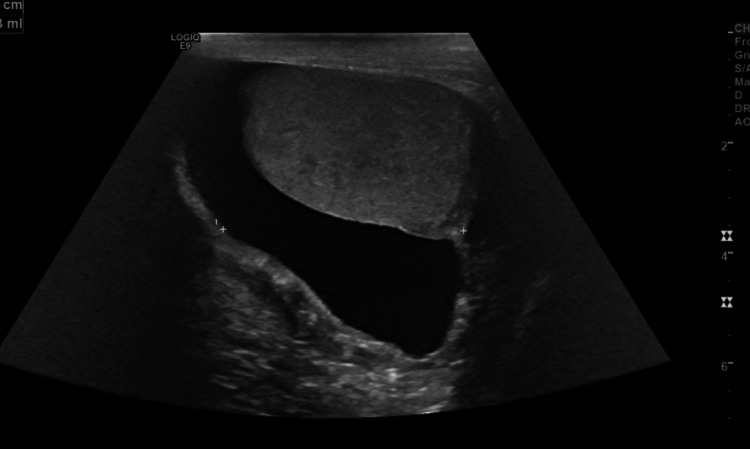
Right homogenous scrotal mass with 79 cc fluid suggestive of hydrocele

Our patient was a 65-year-old male who presented with complaints of progressive painless scrotal swelling for the last five years. Three years prior, he had the scrotal swelling aspirated and a diagnosis of right hydrocoele had been made. Surgery had been recommended as the swelling had persisted. He presented because the swelling, though painless, had slowly increased in size. He had no other urogenital complaints such as dysuria, frequency, or hematuria. Physical exam was consistent with a hydrocoele and urology was consulted. A scrotal ultrasound with Doppler made an impression of a large right-sided hydrocele with an estimated volume of 79 cc. Scrotal ultrasound findings suggestive of hydrocoele are shown in Figure 1.

A right hydrocelectomy was scheduled. Intraoperative findings included a small right hydrocele, thickening, and nodularity of tunica albuginea and spermatic cord. Spermatic cord changes were concerning for malignancy and the nodules were biopsied. Biopsy results revealed scattered atypical cells characterized by multilobate, hyperchromatic nuclei within smooth muscle cells of the spermatic cord. These features were strongly suggestive of well-differentiated liposarcoma with smooth muscle differentiation. Fluorescence in situ hybridization (FISH) performed on the atypical cells showed amplification of MDM2, confirming the histologic impression of liposarcoma. The patient subsequently underwent radical orchiectomy and en bloc scrotal wall excision for liposarcoma. The only postoperative complication was a scrotal abscess and mild wound dehiscence, which was managed with debridement and a course of antibiotics.

Histopathology report was notable for dedifferentiated liposarcoma with heterologous smooth muscle differentiation, tumor dimensions of 11 x 9.5 x 7.5 cm, histologic grade 2 (of 3), and margins narrowly negative for sarcoma. The definitive diagnosis was well-differentiated liposarcoma with heterologous smooth muscle differentiation. Imaging workup after scrotal resection was only remarkable for bilateral sub-centimeter pulmonary nodules, which were indeterminate; otherwise, there was no radiological sign of distant metastasis.

Medical oncology service was consulted, and it was determined that the patient was not a candidate for chemotherapy. Radiation oncology was also consulted as adjuvant radiotherapy was being considered but the recommendation of the radiation oncology team was to withhold radiation and monitor for locoregional recurrence. By means of shared decision-making by the patient and the primary managing team, it was decided that the course of management would be six-monthly follow-ups with imaging surveillance. The patient had no local recurrence during three years of follow-up. Unfortunately, he died due to complications from coronavirus disease 2019 (COVID-19) after three years of follow-up.

## Discussion

Primary malignant spermatic cord tumors can be classified based on their histology and include liposarcoma, rhabdomyosarcoma, leiomyosarcoma, fibrosarcoma, and malignant fibrous histiocytoma. Liposarcoma in turn has five histologic subtypes, which include well-differentiated, myxoid, round cell, pleomorphic, and dedifferentiated. The first two subtypes have a high propensity for locoregional recurrence, and the latter subtypes are more likely to metastasize [3]. Nodal and distant metastasis, however, are less commonly reported and the exact incidence has not been documented in the available literature. Approximately 200 cases have been reported in the literature in English. Our index patient, a 65-year-old, presented with a slow-growing mass that had been present for several years and was thought to be a hydrocele. Sonography was also consistent with a hydrocele. Suspicion for malignancy was raised only after intraoperative findings of nodularity and thickening of the spermatic cord. A definitive diagnosis of liposarcoma was made with histology. This correlates with most other cases in the literature that present in the fifth and sixth decades of life and are often misdiagnosed as inguinal hernias/hydrocele. It was determined that because the mass had been present for several years, the nodules probably started as a well-differentiated liposarcoma that had degenerated to become dedifferentiated. Our patient received radical orchiectomy with en bloc local resection, which is the standard practice. However, postoperative management was challenging due to the absence of clear guidelines regarding adjuvant systemic chemotherapy and radiotherapy. Our patient had narrowly negative margins and the decision not to give adjuvant treatment was based on extrapolation of data from a few available case reports/series reviewed here. We discuss the findings of an exhaustive literature review below.

Role of radiotherapy

A review of the literature revealed six studies [2-7] that provide details on indications for radiotherapy in patients diagnosed with spermatic cord liposarcoma, including modality of administration, dosage, and outcomes on follow-up. Table 1 provides an executive summary of these studies. There was a total of 20 patients.

Table 1 summarizes studies that report indication, dosage for radiotherapy, and outcomes in patients with liposarcoma.

**Table 1:** Studies reporting indication, dosage, and outcome of radiotherapy in patients with liposarcoma

Authors (year), location	Radiation type and number of patients	Follow-up period	Indication for radiation	Radiation dosage	Comments
Cerda et al. (2016), France [2]	Postoperative (n=4)	Median follow-up of 19.5 months	Tumor size >5 cm, intraoperative tumor rupture, margin status	54 Gy	Intensity-modulated radiotherapy was used. No locoregional recurrence during the follow-up period. All patients had cutaneous grade 2 or 3 toxicities from radiotherapy
Ando et al. (2018), Japan [4]	Postoperative (n=1)	1 year at the time of report (10 years planned)	Margin status	60 Gy	No locoregional recurrence within the 1st year of follow-up
Chowdhry et al. (2021), USA [5]	Preoperative (n=3); postoperative (n=4)	Median of 71 months	Anticipated positive margin (preoperative subgroup), inability to achieve wide excision (postoperative subgroup)	52.2 Gy	No locoregional recurrence during follow-up. Complications with wound healing such as chronic sinus tract were encountered in patients who received preoperative radiotherapy
Ballo et al. (2001), USA [6]	Postoperative (n=3)	Median of 9 years	Margin status uncertain	65 Gy	No locoregional recurrence in the follow-up period
Fagundes et al. (1996), USA [7]	Postoperative (n=4)	Median of 63 months	Adenopathy on radiologic lymph node assessment	46.1 Gy	No locoregional recurrence among patients irradiated
Johnson et al. (1978), USA [3]	Postoperative (n=1)	3 years	Local recurrence	60 Gy	Radiotherapy was given after ipsilateral scrotal recurrence 3 years after resection. The tumor recurred on the contralateral suprapubic region 3 years after radiotherapy

Some authors have recommended a combination of surgery and radiotherapy to achieve locoregional control, especially for pleomorphic or round cell histologic subtypes and in situations where the margin status is uncertain due to incomplete tumor removal [3,6]. Among the studies reviewed, prophylactic radiotherapy before or after primary surgery was reported to be effective in achieving locoregional control. There was one case of contralateral side recurrence after radiotherapy as a poorly differentiated tumor [3]. The use of radiotherapy in that single case differs from others in that recurrence after primary surgery had already occurred before radiotherapy was given but this was not effective in preventing further recurrence three years later. The common indications for radiation include positive margin status or inability to achieve a wide resection due to tumor extent [2,4-6]. Other indications mentioned in the literature include intraoperative tumor rupture [2], radiologic evidence of adenopathy [7], and locoregional tumor recurrence after surgery [3].

When comparing pre- versus post-surgical radiation, Chowdhry et al [5] demonstrated the demerits of preoperative radiotherapy as patients who received radiotherapy preoperatively developed complications with wound healing postoperatively. Based on this, they do not recommend preoperative radiotherapy. Ballo et al. [6] have recommended portals of adjuvant radiation covering the most common sites of locoregional relapse.

In a study by Fagundes et al. [7], the indication for postoperative radiotherapy was radiologic evidence of adenopathy. In this series, relapse was more common in patients who received orchiectomy alone. None of the patients who had adjuvant radiotherapy had disease recurrence. These patients were irradiated on the ipsilateral hemiscrotum and iliac and inguinal lymph nodes.

Regarding radiation dosing, the current recommended dose is 60-65 Gy over the course of six weeks [8] The most common cutaneous toxicity of radiation encountered was dermatitis. Dynamic or rotational intensity-modulated radiotherapy was recommended to decrease toxicities due to this [2].

Our patient had wide scrotal excision. Though he had components of dedifferentiation on histology, the margins were negative (distance from sarcoma to closest margin was 0.1 cm) with no evidence of locoregional spread or metastasis, and hence adjuvant radiotherapy was not given.

Role of chemotherapy

Adjuvant chemotherapy has no definitive role in the management of spermatic cord liposarcoma. In the literature, chemotherapy use was only reported in cases with recurrences [8], and its efficacy in preventing locoregional recurrence after primary surgery is yet to be established from published data. Based on this, it was determined that our index patient was not a candidate for chemotherapy.

Follow-up

Follow-up is mandatory given the high propensity for locoregional recurrence. A minimum follow-up period of 36 months to 10 years has been reported in the literature [8] but recurrences can occur decades after the primary surgery. Imaging surveillance is done with interval chest radiographs and CT scans. Our patient remained disease-free at the three-year follow-up.

## Conclusions

Spermatic cord liposarcoma is a rare high-grade tumor with a high propensity for locoregional recurrence often misdiagnosed as hydrocele. We presented a case that was managed with wide excision and follow-up without disease recurrence. We also reviewed the literature on the role of chemotherapy, radiotherapy, and follow-up. A combination of surgery and postoperative radiotherapy is effective in preventing locoregional spread. Data from case reports support this strategy in certain histologic subtypes or when margins are positive after primary surgery. A follow-up period of up to a decade after surgery is recommended.
